# Taking simulation out of its “safe container”—exploring the bidirectional impacts of psychological safety and simulation in an emergency department

**DOI:** 10.1186/s41077-022-00201-8

**Published:** 2022-02-05

**Authors:** Eve Purdy, Laura Borchert, Anthony El-Bitar, Warwick Isaacson, Lucy Bills, Victoria Brazil

**Affiliations:** 1 Gold Coast University Hospital Emergency Department, Southport, Queensland Australia; 2 Faculty of Health Sciences & Medicine, Bond University, Gold Coast, Queensland Australia

**Keywords:** Psychological safety, Teamwork, Translational simulation

## Abstract

**Abstract:**

**Background:**

Simulation facilitators strive to ensure the psychological safety of participants during simulation events; however, we have limited understanding of how antecedent levels of psychological safety impact the simulation experience or how the simulation experience impacts real-world psychological safety.

**Methods:**

We explored the experience of participants in an embedded, interprofessional simulation program at a large tertiary emergency department (ED) in Australia. We engaged in theoretical thematic analysis of sequential narrative surveys and semi-structured interviews using a previously derived framework of enablers of psychological safety in healthcare. We sought to understand (1) how real-world psychological safety impacts the simulation experience and (2) how the simulation experience influences real-world psychological safety.

**Results:**

We received 74 narrative responses and conducted 19 interviews. Simulation experience was both influenced by and impacted psychological safety experienced at the individual, team, and organizational levels of ED practice. Most strikingly, simulation seemed to be an incubator of team familiarity with direct impact on real-world practice. We present a model of the bidirectional impact of psychological safety and simulation within healthcare environments.

**Conclusion:**

Our model represents both opportunity and risk for facilitators and organizations engaging in simulation. It should inform objectives, design, delivery, debriefing, and faculty development and firmly support the situation of simulation programs within the broader cultural ethos and goals of the departments and organizations.

**Supplementary Information:**

The online version contains supplementary material available at 10.1186/s41077-022-00201-8.

## Introduction

Discourse related to psychological safety within the simulation community has centred on how facilitators can create a “safe container” for participants, but this narrow emphasis limits the potential power of a critical teamwork concept [1]. The next step to inform our approach, as we seek to improve the performance of healthcare teams, is to understand how psychological safety leaks into, and out of, the container of simulation.

Psychological safety—“a shared belief held by members of a team that the team is safe for interpersonal risk taking”—informs simulation facilitators’ current approaches and also has real-world implications for teams [1, 2]. Simulation facilitators have diligently focused on fostering psychologically safe learning environments. Many employ pre-briefings, rapport building, fiction-contracts, and a variety of other tools in hopes of creating and maintaining space for interpersonal vulnerability and collective learning, with variable success [1, 3–5]. But the concept of psychological safety originates and extends well beyond the walls of the simulation space. Psychological safety is directly linked to real-world team performance through the impact of interpersonal risk taking on speaking up behaviors, teamwork behaviors, and team learning [2, 6]. Within healthcare, a recent systematic review of psychological safety identified 13 enablers at the individual, group, and organizational levels [7]. Yet, interventions designed to improve real-world psychological safety, some of which include simulation, have had varied success [8]. To have the most impact as a community of practice, it is time to link our understanding of psychological safety with real-world team performance rather than limit it to the simulation session in front of us.

Driven by our experiences as simulation facilitators across a variety of healthcare contexts and informed by acknowledgement in the original psychological safety literature that “practice fields” may be antecedents to team psychological safety [9], we designed a study to better understand the interplay between simulation and psychological safety. Specifically, we were interested in exploring both:

- How real-world team psychological safety influences simulation experience

and

- How simulation impacts real-world team psychological safety

## Methods

We engaged in a theoretical thematic analysis of sequential narrative surveys and semi-structured interviews using a previously derived framework of enablers of psychological safety in healthcare [7, 10]. This was part of a larger study related to psychological safety in the emergency department (ED), and it was approved by the Gold Coast Hospital and Health Service Research Ethics Committee (HREC/2020/QGC/60733).

### Context

The study took place in the ED of a large tertiary hospital on the Gold Coast of Australia. The ED census is 155,000 patients each year, including paediatrics and major trauma, and the unit is staffed by over 300 nurses and approximately 120 doctors. Simulation-based training is well embedded in ED practice, with weekly simulation based educational session for medical trainees and nursing staff. The weekly interprofessional simulation program is attended by 4–6 emergency medicine registrars and 8–10 registered nurses. Scenarios are developed based on common and important ED presentations, and responsive to current educational needs and quality and safety issues within the ED. The debriefing, conducted by trained faculty, is aligned with the “Promoting Excellence and Reflective Learning in Simulations” framework, with a focus on clinical issues, teamwork, and the ED system [11].

### Data collection

Nurses, registrars, and emergency consultants were invited to participate. Eligible participants received an email link to the survey (Additional file 1) and a follow-up reminder email. Of note, this survey also included a quantitative analysis of psychological safety as part of the larger study. Participants completing the survey were invited to participate in interviews, and additional purposive sampling across experience levels and professions was used to identify additional interviewees. Survey completion was not mandatory for participation in interviews. The narrative survey and interview guide were piloted with ED staff before circulation and use. The interviews were conducted via phone or in person by EP. Interviews were recorded and transcribed using NVivo then checked by EP and LBo.

### Data analysis

Narrative survey responses and interview data were analyzed using deductive thematic analysis [10]. They were coded by EP and LBo in Nvivo using a previously derived framework for psychological safety in healthcare with 13 codes at the individual, team, and organizational levels (Table 1) [7]. EP and LBo met throughout this process to compare coding and themes. VB was available to mediate any discrepancies in this process. Throughout the process, LBo and EP kept reflexive journals and positioning was frequently discussed at team research meetings. They reflected on how their involvement as simulation facilitators and participants impacted their interpretation of the data and attempted to challenge those perspectives by finding opposing examples in the data. The analysis was shared first with other members of the research team who had full access to the data, then members of the medical and nursing teams. Feedback on our analysis was sought and incorporated at each of these stages. The full dataset generated is not publicly available to maintain participant confidentiality, but de-identified data may be available from the corresponding author on request.

**Table 1:** Framework for enablers of psychological safety in healthcare adapted from O’Donovan et al. [7]

Individual	Team	Organizational
-Professional responsibility -Individual differences	-Leader behavioural integrity -Status, hierarchy, and inclusiveness -Peer support -Leader support -Change oriented leadership -Familiarity with leader -Familiarity with team members	-Safety culture -Continuous improvement culture -Organizational support -Familiarity across teams

### The study team

Our team is oriented towards social constructivism and informed by our experiences designing, delivering, and debriefing simulation across a variety of healthcare contexts. We are leaders in translational simulation [12, 13] and are particularly interested in the impact of simulation on team relationships and culture [14, 15]. EP and LBo were the authors primarily involved in data collection and analysis. EP is an emergency physician and applied anthropologist with background in organizational culture in healthcare teams. At the time of the interviews, she was hired, but not yet employed or in her first month of employment, at the Gold Coast University Hospital (GCUH) ED as a research and clinical fellow. She had previously worked at GCUH so had strong contextual understanding of the organization and simulation program. LBo and AE are medical students with experience as simulation participants but limited exposure to the ED. VB is the medical director of the Simulation Service at GCUH and connected to the international healthcare simulation community. WI is a consultant in the ED and LBi is a clinical nurse educator, both are faculty in the simulation program.

## Results

A total of 35/300 nurses, 20/60 registrars, 14/50 consultants, and 3 nurse educators completed the surveys; this reflects a response rate of 17.4%. EP conducted 19 interviews (9 nurses, 9 registrars, 1 consultant) with a mean duration of 17.6 min (7:14–33:53 min). Interviews were with staff at varying experience levels and length of time working at GCUH.

**Fig. 1:**
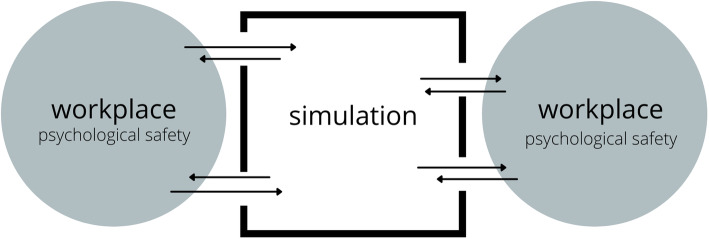
Bidirectional flow of psychological safety into and out of simulation

We explored the bidirectional impact of simulation on psychological safety and psychological safety on simulation (Fig. 1) at the individual, team, and organizational levels.

### Individual

At the individual level, participants found simulation was both impacted by and had real impacts on confidence, a known key individual difference in mediating psychological safety [16].

A small group of participants were very confident and keen to participate in simulation. One nurse said she was “Stoked [to be picked for simulation]. [I] Enjoy the challenge and take it as a great learning and training opportunity”. This growth mindset, as a starting place, well positioned some participants to engage with the interpersonal risks of simulation. Most other participants fell into the category of feeling some anxiety around simulation but to a degree that did not necessarily prohibit learning. Like one registrar who said, “[simulation makes me] anxious because they are quite high stress but also look forward to it as always great learning” or another nurse who was “nervous but excited to be challenged”. This group often commented on the efforts that the facilitators took—such as distributing pre-readings and thoughtful pre-briefings—as helpful in making the transition from anxious to engaged. A much smaller but important proportion of participants had a pathologic degree of anxiety related to simulation that likely impaired their ability to take any interpersonal risks during simulation. Some in this group described visceral reactions including diarrhea or nausea. Others purposefully scheduled the days of simulation off work. Many in this group could describe in vivid detail a negative experience related to simulation from early in their training. This spectrum of individual confidence in skills and with simulation presents a challenge for simulation facilitators.

Simulations also had the ability to impact real-world psychological safety through the development or destruction of confidence in personal knowledge and skills. For example, registrars shared that simulations “improve clinical skills and confidence in managing critical patients” and serve as a “major confidence booster when dealing with high acuity problems.” One nurse described feeling “more confident using the airway equipment” after participating in a simulation as the airway assistant. At the same time, we saw examples of where design, delivery, and debriefing decisions negatively impacted confidence with real-world consequences. For example, one nurse commented on how being put into a role that she would not usually be in, negatively impacted her confidence.

They put me in the drug role position and I wasn’t even familiar with that role. It was totally negative because everybody was having fun except for me…I was out of my depth and I knew it…I gave drugs that I wouldn’t usually be giving and it made me feel stupid.– Nurse (interview 1)

Similarly, a registrar shared how such a negative experience could impact their experience in the workplace.

If I felt I did poorly in a sim it would affect me for the rest of the day and sometimes multiple. I felt like I was mentally and emotionally overwhelmed and wasn’t able to concentrate for the rest of my shift. Registrar (survey)

### Team

At the team level, psychological safety both impacted simulation experience and was impacted by the simulation experience—a bidirectional effect largely driven by familiarity. Pre-existing familiarity with the team members participating in the simulation modulated the learning for some but more dramatically was a key outcome of the simulation for participants. Leader familiarity, leader behavioral integrity, and inclusiveness were also important outcomes of the simulations for teams.

For example, one nurse described his learning experience in simulations as being different depending on the level of familiarity he had with the other participants he entered the simulation with.

You may learn better from the scenario because you are not thinking, ‘I haven’t worked with this person before’ and trying to build that relationship. There is only so much you can comprehend. – Nurse (interview 18)

He went on to describe that for complex scenarios having a team that you know well allows you to engage in problem solving more fully whereas for more simple scenarios an unfamiliar team may be acceptable because there are fewer interpersonal challenges to navigate. Other participants highlighted the importance of the nature of the pre-existing relationships and familiarity with the simulation faculty as particularly relevant to their learning experience both positively and negatively.

The impact of simulation on the development of familiarity with team members and team leaders was the most central finding of our study as it relates to how simulation impacts psychological safety on the floor. Simulation was an incubator of familiarity and acted as a magnifying glass on leader behavioral integrity. It was clear that participants viewed simulation as a place where relationships are forged, with both positive and negative consequences. Many described feeling more empowered to take interpersonal risks on the ED floor after working with medical staff in simulation. Like this nurse who wrote,

I felt that I could be open with my colleagues in this scenario [geriatric patient with bradycardia] and would have been able to speak up had I felt that intubation was not in the patient's best interests. I believe this simulation strengthened my relationships with the registrars involved and it confirmed that they would respect my opinion in a similar scenario. – Nurse (survey)

The impact on familiarity seemed particularly relevant for new hires and those training in new roles. They worried about it negatively impacting their reputation but also recognized it as a potentially positive space to build meaningful relationships. One registrar (interview 12) who was new to the department said, “there’s a lot of fear about being out of my depth and not really knowing what to do and that being on display for everyone I work with.” While a nurse new to the hospital highlighted how it enabled her to become more familiar with her team, “I’ve come to a new team and it gives me an opportunity to see how they work, how they want me to work, and what works best.”

In particular, and quite specific to the context of our simulation program, registrars felt that it was a place that they could build credibility with the nursing staff. For example, one registrar said, “[it’s an opportunity to] instill faith in your team and make sure that they know that you know what you are actually doing…” (interview 11). At the same time, nurses found that it was a place that they could get to know the leadership styles of the registrars. Taking it even further a more senior nurse saw it as their opportunity to prospectively shape registrars’ leadership approaches.

Sometimes we might just assume the registrars are always really good, but it’s good to support the junior doctors as they step up. You can see their growth….and build a connection with those registrars you are working with. – Nurse (interview 4)

But along with the potential positives for psychological safety at a team level comes a real risk of negative consequences. There were not many, but some important examples, of when simulation negatively impacted real-world relationships. One nurse responded, “the simulation I was involved in was a horrible experience…we were not respected by the team leading doctor” or another who shared, “if I make a mistake during simulation it sets a negative tone for the rest of the shift.” Though we were not able to interview these participants to learn more, it is reasonable to extrapolate that such negative experiences, or similar that risk being underreported in our sample, have important consequences for teams on the ED floor.

### Organization

Organizational factors influence psychological safety entering the simulation. Rostering choices impacted the ability for some to participate which created tension and hierarchy between some staff. Even worse in rare occasions without appropriate cover, simulation was perceived to potentially negatively impact patient safety on the ED floor. One nurse wrote, “daily checks weren’t complete and surrounding team members weren’t supported on the floor.” More positively, at the organizational level the simulation program was seen as living evidence of an organizational commitment to continuous improvement and safety culture. One registrar suggested,

“It’s the feeling that we are trying to push the envelope…like the getting to CT simulations and improving the times of getting trauma patients scanned…it feels nice, like you are working somewhere that does something good.” – Registrar (Interview 3)

Though not directly related to the ED simulations that were the focus of this study some participants did comment on how interdepartmental simulations increased their understanding of other departments’ roles and improved relationships across traditional organizational lines. We heard from participants that simulation modeled how to have conversations about improvement which some started incorporating into performance conversations with colleagues on the floor in the form of after-action reviews or hot debriefs.

The simulation program has greatly improved my own teamwork skills. I have developing awareness for higher order communication strategies that incorporate team briefings…and the use of hot debriefing techniques. – Consultant (survey)

These types of comments reflect that the simulation process itself magnifies important organizational values related to continuous improvement and teamwork.

## Discussion

Our findings suggest that simulation is not actually a container at all—at least not an airtight one. Rather, it is a bidirectionally leaky construct (Fig. 1). The antecedent psychological safety of teams participating in simulation has major influence on their simulation experience, which in turn impacts their experience of safety in the real working environment. This reconceptualization adds weight to our responsibility as simulation facilitators and extends that responsibility to clinical leaders in healthcare teams. The model also suggests simultaneous risk and opportunity for organizations. A recognition of the bidirectionality of psychological safety in simulation must shape the way we situate, conceptualize, design, deliver, and debrief simulation in healthcare.

### Minding what leaks in (Fig. 2)

Preceding individual factors impacted participants’ experience in the simulation container. Most notably, prior experiences with simulation and differing levels of personal confidence were dominant to perceptions related to interpersonal risk taking which is in keeping with other studies [16, 17]. Many practices used by facilitators to build and maintain psychological safety are appropriately in keeping with attending to these needs [1, 3, 5, 18]. A Delphi study of simulation educators and a recent systematic review of pre-simulation and pre-briefing also highlighted the need for facilitators to tailor efforts to build psychological safety to these individual factors [19, 20].

**Fig. 2:**
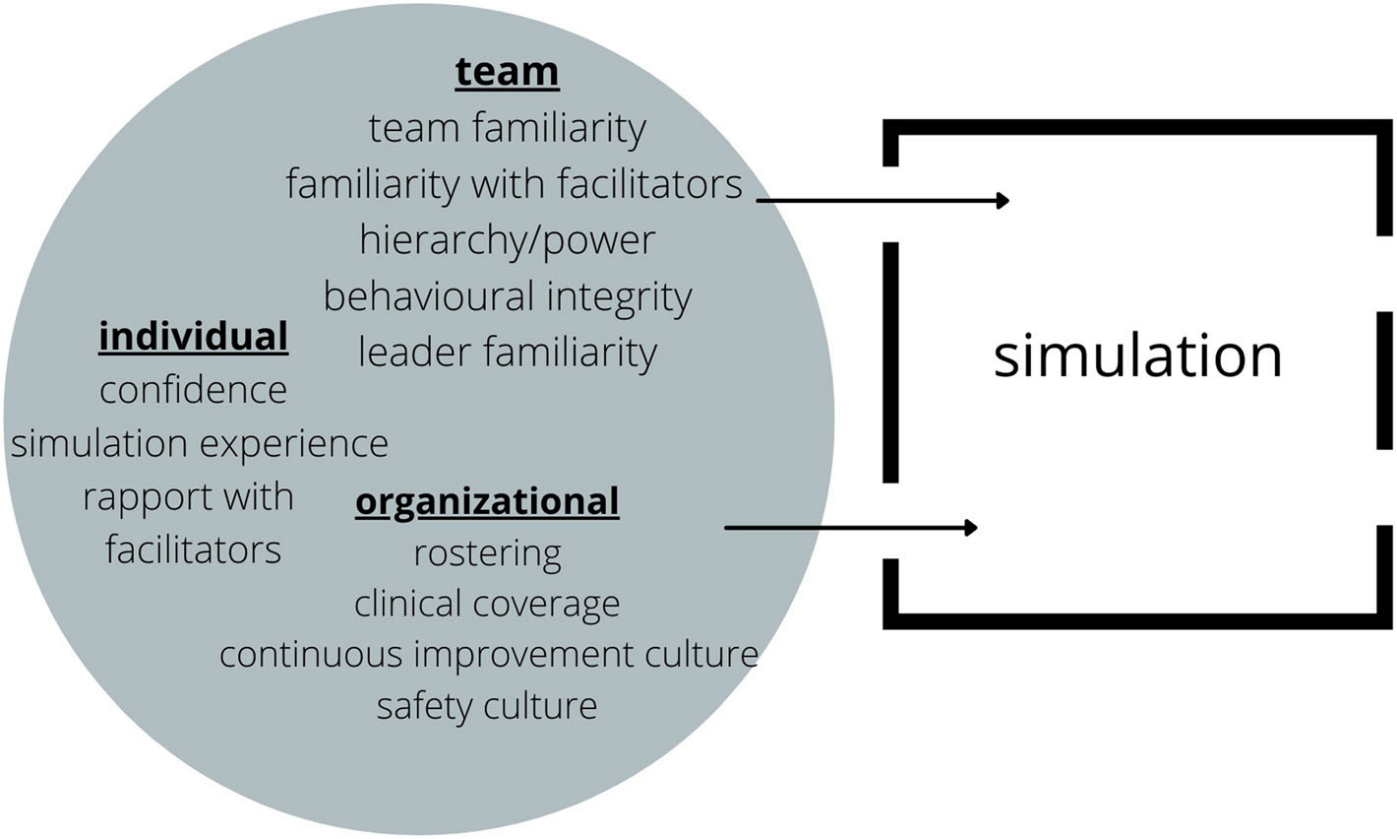
The flow of psychological safety into simulation

Though less dominant in our data, pre-existing familiarity with team members, relationship with facilitators, and organizational factors were relevant to safety and learning. This is hardly surprising, but we worry that these issues might be underappreciated by the simulation community. Seminal articles that review the concept of creating psychological safety in simulation do not adequately address the importance of these pre-existing interpersonal realities [1, 21]. Our study adds empirical evidence and weight to recently published editorial papers that theorize the importance of these additional factors [3, 18, 22]. It is likely that groups with high degrees of pre-existing of familiarity and psychological safety will be well positioned to take significant interpersonal risks. Unfamiliar but neutral groups will benefit from more attentive introductions and intra-participant rapport building in the pre-briefing blended with simpler case design. Groups with low levels of psychological safety or negatively rooted familiarity may struggle to be safe enough for meaningful engagement despite efforts to build safety from facilitators. More often, groups will be heterogeneously comprised along this spectrum. Similarly, the pre-existing degree of credibility and temperature of relationships of facilitators with participants before entering the simulation event may positively or negatively impact psychological safety and learning. This is in keeping with education literature that suggests a correlation between teacher credibility (competence, trustworthiness, and caring) and student outcomes [23]. Organizational factors including commitment to the improvement ethos and practical considerations related to rostering are also relevant to participants’ perceived psychological safety in simulation.

Drawing on our research, we have several practical suggestions for facilitators and departments looking to broaden their awareness of the pre-existing factors that impact psychological safety in simulation (Table 2). Thoughtfully predicting what might be harmful and capitalizing on what might be helpful at the individual, team, and organizational level will empower us to best manage leaks into the “container” of simulation.

**Table 2:** Practical tips for managing the bidirectional flow of psychological safety into and out of simulation

*Minding the leaks in*	*Shaping the leaks out*
**Diagnose** and manage the team dynamics before even entering the room. Prior experience with simulation, team familiarity, hierarchy, pre-existing relationships, and power dynamics are relevant to the simulation experience.	**Stop** saying, “what happens in simulation, stays in simulation” [25]. It just isn’t true. We show that simulation impacts ideas, relationships, and judgements participants have about colleagues, their organizations, and themselves.
**Reflect** on your own positioning as a facilitator. Your credibility and pre-existing relationships with participants matter. If you don’t foster psychological safety outside of the simulation room, you shouldn’t be a facilitator in it.	**Continue** employing traditional ways of building, maintaining, and repairing psychological safety in the simulated environment [1, 3, 18, 21, 24]. This likely results in a significant leak of familiarity, confidence, leadership behaviours, and trust back into the working environment.
**Commit** at an organizational level to the process. To be most effective, simulation will be a manifestation of an improvement ethos not an isolated event.	**Start** overtly debriefing around concepts related to psychological safety. Name and explore ideas like familiarity, role understanding, supportive leadership, trust, inclusiveness, belonging, speaking up, and confidence—or what gets in the way of them.

### Shaping what leaks out (Fig. 3)

Just as psychological safety leaks in, our findings provide evidence that it also leaks out. For our ED teams, simulation was an incubator of psychological safety at the individual, team, and organizational levels with translation back to the working environment, with the potential for both positive and negative impacts. These findings support previous research and theoretical discussions that position simulation as a moment of cultural compression impacting real world values, beliefs, and relationships [9, 14, 15, 22, 24]. With this reality, however, comes significant responsibility. Facilitators should aim to deliberately shape the outflow of psychological safety. We suggest three practical steps for doing so (Table 2).

**Fig. 3:**
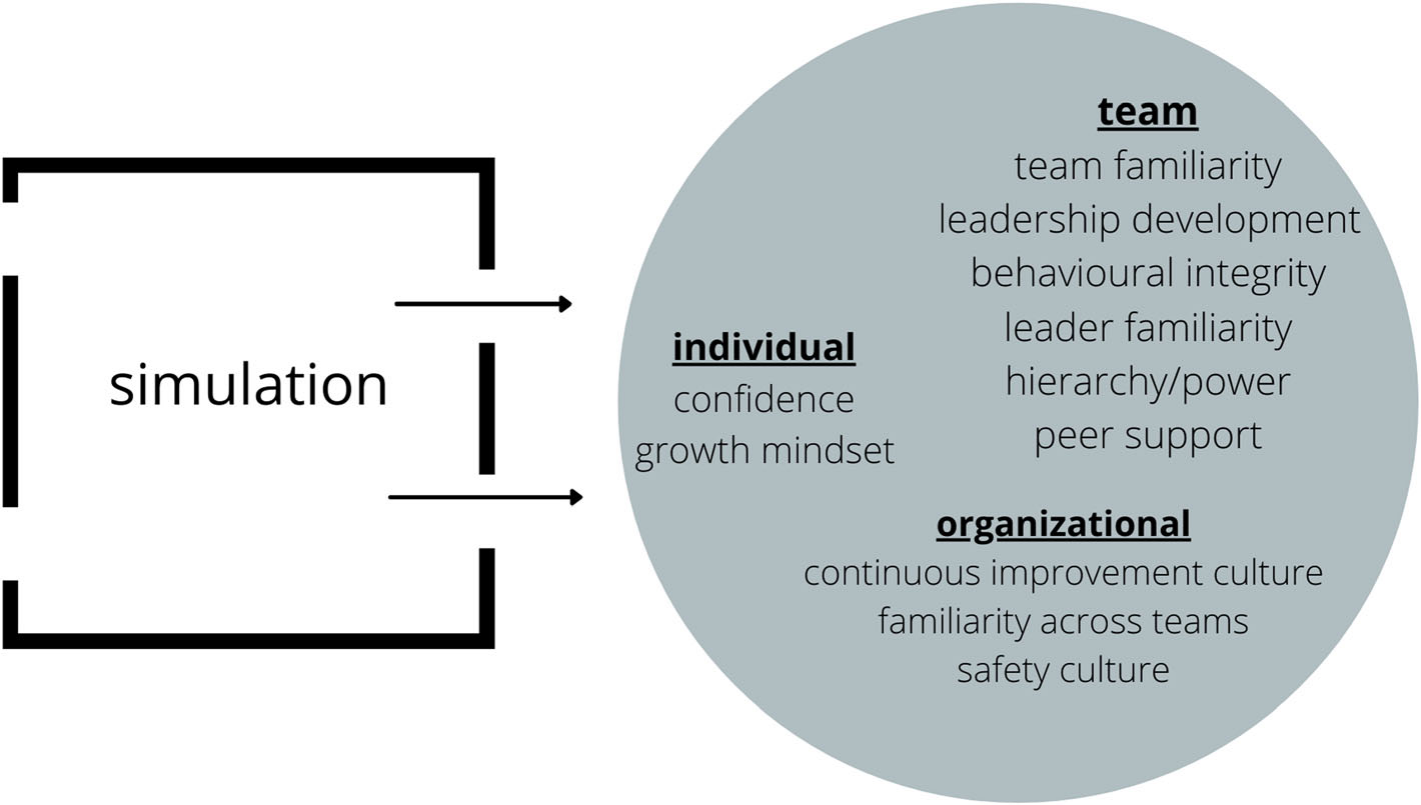
The leak of psychological safety out of simulation

The “leak out” is particularly exciting for those looking for tangible ways to shape team affect and organizational culture. The opportunity that it presents to efficiently enhance familiarity for teams is particularly attractive. Departmental leaders should look to embedded simulation programs as an opportunity to bolster psychological safety but must also be aware of the real risks associated with programs that are problematically designed, delivered, or debriefed. Ongoing reflection, feedback, and analysis of the impact of simulation on less frequently measured outcomes related to organizational culture, like psychological safety, should become the norm within our organizations to ensure that simulation programs are have maximum benefit and minimum harm.

### Implications and future research

At a local level we are using this model to reflect on and refine our simulation design, delivery, debriefing, and faculty development. As usual, we are left with more questions than answers. Future research could explore how to identify which teams are positioned to benefit most from simulation based on their pre-existing levels of psychological safety. We are also interested in understanding the dose and best participant targets to efficiently maximize impact. Others might explore specific strategies and approaches that facilitators should take to understand dynamics before the simulation or to further explore the relevance of credibility and optimal positioning of facilitators. Furthermore, we encourage the application of this bidirectional model across simulation contexts to refine and identify the most relevant, generalizable, and actionable aspects for your team and for the broader simulation community.

### Limitations and realties

We have a natural leaning towards translational simulation and seek to understand the impact of simulation activities through an organizational culture lens. We are not inclined to ask “if” that happens but rather find ourselves asking “how” it does. Unavoidably, our research questions and interpretation are informed by our experience as simulation facilitators and our research experience goes on to inform our approach to simulation design, delivery, and debriefing. We recognize that our proximity impacts the questions we ask, data we collect, and interpretation we engage in. For this study, we felt the benefits of that proximity outweighed the risks since we were interested in deep understanding, that requires contextual knowledge, rather than any form of evaluation of the “success” of the program. To mitigate power imbalances EP, as a relative junior within the organization, conducted the interviews. To help widen our lens we had members of our research team who are not usually embedded in the department or simulation team involved in data analysis phases.

There are key realities to keep in mind when interpreting our results. Our survey response rate was low. However, these findings were further characterized in the interviews. More in-depth exploration of people with negative experiences would be a next step to enhance our simulation community’s understanding of the implications in the workplace. Furthermore, we explored a well-established, departmentally embedded, simulation program that is coordinated and run by experienced facilitators. This is differently oriented to simulation in purely educational contexts and will have any number of differences to other translational simulation programs. As such, the specific findings may not be relevant across contexts but we are optimistic that the model proposed for the bidirectional impact of psychological safety will be universally translatable.

## Conclusion

In an embedded ED simulation program, individual, team, and organizational domains of psychological safety impact the simulation experience and are shaped by the simulation experience. We propose a model that simultaneously builds on and challenges the “safe container” [1] by highlighting the bidirectional impact of psychological safety and simulation. It should inform objectives, design, delivery, debriefing, and faculty development—especially for those involved in translational simulation programs. To maximize impact, simulation facilitators can use this model to reframe the way they understand the important teamwork theory and organizations can use it to situate simulation within their broader priorities.

## Supplementary Information

**Additional file 1.** Survey questions.

**Additional file 2.** Interview questions.

## Data Availability

The full dataset generated is not publicly available to maintain participant confidentiality, but de-identified data may be available from the corresponding author on request.
