# Supplementary material for: Taking simulation out of its “safe container”—exploring the bidirectional impacts of psychological safety and simulation in an emergency department
Source: Adv Simul (Lond). 2022 Feb 5;7:5. doi: 10.1186/s41077-022-00201-8 (PMC8818167; doi:10.1186/s41077-022-00201-8)
Supplement: Supplementary file 1 — Additional file 1. Survey questions. [file 41077_2022_201_MOESM1_ESM.pdf]

Exploring the relationship between psychological safety in the workplace and psychological safety in simulation based educational sessions for emergency department doctors and nurses

About you

What is your role in ED?

How many years have you worked at GCUH or Robina ED?

How many ED simulation sessions have you attended in the last 12 months?

<5, 5 – 10, more than 10

Psychological safety scale questions

Please rate your level of agreement with the following statements

- If you make a mistake in this team, it is often held against you.
- Members of this team are able to bring up problems and tough issues.
- People on this team sometimes reject others for being different.
- It is safe to take a risk on this team.
- It is difficult to ask other members of this team for help
- No one on this team would deliberately act in a way that undermines my efforts
- Working with members of this team, my unique skills and talents are valued and utilised

7 point scale from very accurate to very inaccurate

Narrative Survey Questions

1. Describe the last simulation activity you were involved in. Did participating in this activity impact your day to day practice in the ED? If so, how?
2. Describe how your involvement in the simulation program over months/years impacts your work in the emergency department?
3. Describe how simulation impacts your relationships with members of the ED team?
4. When you find out you will be participating in a simulation session how do you feel? Why?
5. Describe a time you were worried about a patient in the ED. Were you able to raise those concerns? Why/why not.
6. Describe how hierarchy affects your experience working in the ED.
7. Do you feel valued as a member of the ED team? Why or why not?

Thank you for participating in our study survey. We appreciate your time.

As part of the study we are also asking ED doctors and nurses to participate in short interviews. These are intended to build on the survey responses and build on relationships, teamwork and learning between emergency department providers, and their experiences with the ED simulation program.

Participation is voluntary.
If you consent to be contacted for more information please provide your contact details here (link https://forms.gle/9taMa9Pjrz77L3CB7)

This takes you to a separate link so that your survey responses remain anonymous
