# Supplementary material for: Taking simulation out of its “safe container”—exploring the bidirectional impacts of psychological safety and simulation in an emergency department
Source: Adv Simul (Lond). 2022 Feb 5;7:5. doi: 10.1186/s41077-022-00201-8 (PMC8818167; doi:10.1186/s41077-022-00201-8)
Supplement: Supplementary file 2 — Additional file 2. Interview questions. [file 41077_2022_201_MOESM2_ESM.pdf]

Exploring the relationship between psychological safety in the workplace and psychological safety in simulation based educational sessions for emergency department doctors and nurses

Interview Questions

These will be subject to minor change based on findings in the narrative survey data.
Themes are based on a recent systematic review.

Thank you for meeting with me today. We will spend about 45 minutes discussion your experience working in the ED and with our simulation program. This interview will be recorded and transcribed then this recording will be deleted. During that process the interviews will be de-identified. You will be able to review that transcript by request. Any questions before we get started?

General

-Tell me about your role in the ED
-Tell me about your experience with simulation in the emergency department
- explore negatives
-explore positives
-What do you feel the main purpose of the weekly simulation (registrar simulation) program is?
-What do you feel is the greatest value add of the weekly simulation program?
-What would you change about the registrar simulation program?
- Are there any real or potential harms associated with the weekly simulation program?

-When you participate in simulation activities…
-what makes you feel comfortable
-what makes you feel uncomfortable
-tell me about an experience when you felt uncomfortable
-tell me about a time that you made a mistake while participating in sim?

-When you are working in the ED
-what makes you feel safe
-what makes you feel uncomfortable – tell me about a time when you feel uncomfortable in the ED
-what makes you feel like you can take interpersonal risks (i.e. share if you think something is wrong or challenging a plan that your colleague has come up with)

Theme: Support/ Theme: Familiarity with Colleagues/ Theme: Status/Hierarchy/Inclusiveness

Tell me about your relationships with your ED colleagues?

-how are these relationships built? (individual/departmental/organizational)

-what threatens these relationships? (individual/departmental/organizational)

Does simulation impact your relationships with ED colleagues? How?

As a department how do you think we might support strong relationships?

Tell me about the hierarchy in the ED?

-why do you think this hierarchy exists?

-are there benefits/drawbacks?

Theme: Priority for Patient Safety/ Theme: Improvement for learning orientation

How does the emergency department approach patient safety?

-what are the barriers to providing safe care

-what supports your ability to provide safe care

How does the emergency department approach quality improvement?

-what are the barriers to providing excellent care?

-what supports your ability to provide excellent care?

How does simulation impact patient safety?
